# Supplementary material for: A longitudinal pilot study in pre-menopausal women links cervicovaginal microbiome to CIN3 progression and recovery
Source: Commun Biol. 2025 Jun 6;8:883. doi: 10.1038/s42003-025-08328-w (PMC12144234; doi:10.1038/s42003-025-08328-w)
Supplement: Supplementary file 1 — Supplementary Information [file 42003_2025_8328_MOESM1_ESM.pdf]

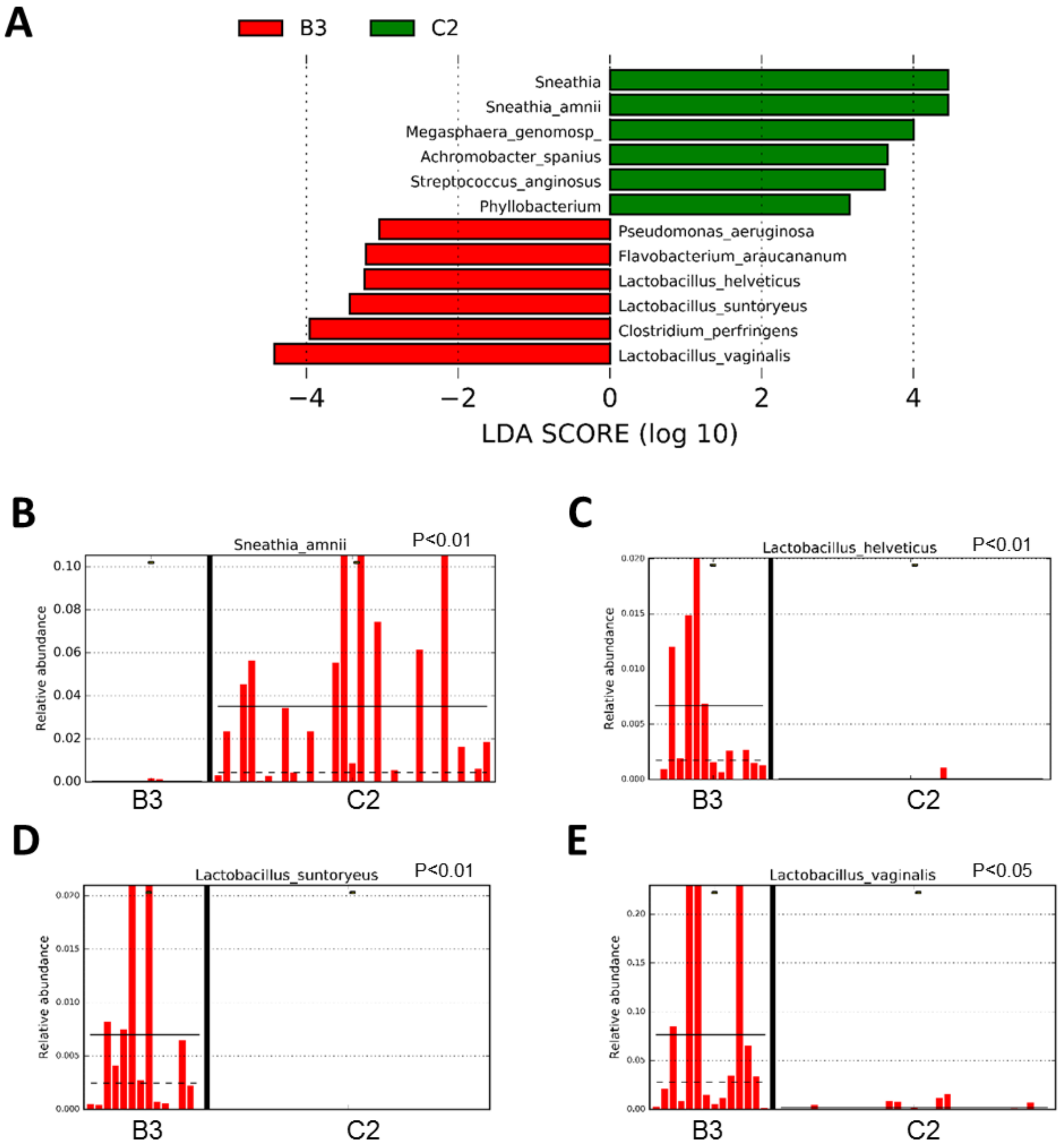

Supplementary Figure 1 – Microbial biomarkers discovery in persistently HPV positive women with normal cytology (B3) vs CIN3 diagnosed women (C2) by LEfSe analysis. (A) Histogram of the LDA scores computed for features differentially abundant in each subgroup (C2 vs B3). Relative abundance counts of Sneathia amnii (B) which was found to be significantly overexpressed in C2, whereas Lactobacillus helveticus (C), Lactobacillus suntoryeus (D) and Lactobacillus vaginalis (E) were enriched in B3 (Welch's t-test). The threshold for the logarithmic LDA score was 2.0. Abbreviations: B3: subgroup of B, including persistently hr-HPV positive (over 6 years) women with normal cytology results; C2: subgroup of C, including CIN3 diagnosed women; LDA score: Linear discriminant analysis score. LEfSe: Linear discriminant analysis effect size.
