# Supplementary material for: A longitudinal pilot study in pre-menopausal women links cervicovaginal microbiome to CIN3 progression and recovery
Source: Commun Biol. 2025 Jun 6;8:883. doi: 10.1038/s42003-025-08328-w (PMC12144234; doi:10.1038/s42003-025-08328-w)
Supplement: Supplementary file 2 — Description of Additional Supplementary Files [file 42003_2025_8328_MOESM2_ESM.pdf]

Description of Additional Supplementary Files

File Name: Supplementary Data 1

Description: Operational taxonomic unit (OTU) table with read counts for all samples in the ARTISTIC cohort. Each row corresponds to a sample and each column to an OTU named after the corresponding annotated species. Sample names include information on the sequencing runs performed in the project. Suffixes such as '_merged' in the sample names indicate three sequencing runs have been merged together in a file, '_v1' indicate only one sequencing run has been used for analysis. No suffix on the sample indicates only two sequencing runs merged together for subsequent analysis.
