# Supplementary material for: A longitudinal pilot study in pre-menopausal women links cervicovaginal microbiome to CIN3 progression and recovery
Source: Commun Biol. 2025 Jun 6;8:883. doi: 10.1038/s42003-025-08328-w (PMC12144234; doi:10.1038/s42003-025-08328-w)
Supplement: Supplementary file 4 — Reporting Summary [file 42003_2025_8328_MOESM4_ESM.pdf]

nature portfolio

Corresponding author(s): DAPR COMMSBIO-24-7085A

Last updated by author(s): 28/05/2025

Reporting Summary

Nature Portfolio wishes to improve the reproducibility of the work that we publish. This form provides structure for consistency and transparency in reporting. For further information on Nature Portfolio policies, see our Editorial Policies and the Editorial Policy Checklist.

Statistics

For all statistical analyses, confirm that the following items are present in the figure legend, table legend, main text, or Methods section.

n/a	Confirmed	
☐	☒	The exact sample size (*n*) for each experimental group/condition, given as a discrete number and unit of measurement
☐	☒	A statement on whether measurements were taken from distinct samples or whether the same sample was measured repeatedly
☐	☒	The statistical test(s) used AND whether they are one- or two-sided<br>*Only common tests should be described solely by name; describe more complex techniques in the Methods section.*
☒	☐	A description of all covariates tested
☐	☒	A description of any assumptions or corrections, such as tests of normality and adjustment for multiple comparisons
☐	☒	A full description of the statistical parameters including central tendency (e.g. means) or other basic estimates (e.g. regression coefficient) AND variation (e.g. standard deviation) or associated estimates of uncertainty (e.g. confidence intervals)
☐	☒	For null hypothesis testing, the test statistic (e.g. *F*, *t*, *r*) with confidence intervals, effect sizes, degrees of freedom and *P* value noted<br>*Give P values as exact values whenever suitable.*
☒	☐	For Bayesian analysis, information on the choice of priors and Markov chain Monte Carlo settings
☐	☒	For hierarchical and complex designs, identification of the appropriate level for tests and full reporting of outcomes
☒	☐	Estimates of effect sizes (e.g. Cohen's *d*, Pearson's *r*), indicating how they were calculated

Our web collection on statistics for biologists contains articles on many of the points above.

Software and code

Policy information about availability of computer code

Data collection	No software was used
Data analysis	LefSe analysis was conducted on the publicly available software on Harvard University’s Galaxy instance (http://galaxy.biobakery.org/). All staststical tests were performed in R 3.6.1.

For manuscripts utilizing custom algorithms or software that are central to the research but not yet described in published literature, software must be made available to editors and reviewers. We strongly encourage code deposition in a community repository (e.g. GitHub). See the Nature Portfolio guidelines for submitting code & software for further information.

Data

Policy information about availability of data

All manuscripts must include a data availability statement. This statement should provide the following information, where applicable:

- Accession codes, unique identifiers, or web links for publicly available datasets
- A description of any restrictions on data availability
- For clinical datasets or third party data, please ensure that the statement adheres to our policy

The OTU table created and used for each analytical step is included as Supplementary Data 1. The 16s rDNA raw sequencing data for this study have been deposited in the Genome Sequence Archive (https://ngdc.cncb.ac.cn/gsa/) under the BioProject PRJCA039846.

Research involving human participants, their data, or biological material

Policy information about studies with human participants or human data. See also policy information about sex, gender (identity/presentation), and sexual orientation and race, ethnicity and racism.

Reporting on sex and gender	No sex and gender-based analysis was performed as this study recruited only biological women.
Reporting on race, ethnicity, or other socially relevant groupings	All selected women were Caucasian and pre-menopausal (20-53 years).
Population characteristics	Sixty-five women were selected from the ARTISTIC trial, which compared screening outcomes using cervical cytology alone versus cytology plus HPV testing over two rounds, three years apart, as described in detail previously (Kitchener et al 2009 DOI: 10.1016/S1470-2045(09)70156-1). All selected women were Caucasian and pre-menopausal (20-53 years) with hrHPV genotyping results available for each screening round. Samples from six women (n=18) were excluded due to inadequate read coverage (< 10,000 reads per sample). The final analysis looked at 59 women (samples, n=177).
Recruitment	See above
Ethics oversight	The use of the ARTISTIC samples in this study have been approved by the NRES Committee Southeast Coast – Brighton and Sussex (Study title: Long-term follow-up of ARTISTIC cervical screening trial cohort) with the REC reference: 14/LO/0627 IRAS project ID: 153311.

Note that full information on the approval of the study protocol must also be provided in the manuscript.

Field-specific reporting

Please select the one below that is the best fit for your research. If you are not sure, read the appropriate sections before making your selection.

☒ Life sciences ☐ Behavioural & social sciences ☐ Ecological, evolutionary & environmental sciences

For a reference copy of the document with all sections, see nature.com/documents/nr-reporting-summary-flat.pdf

Life sciences study design

All studies must disclose on these points even when the disclosure is negative.

Sample size	Sixty-five women were selected from the ARTISTIC trial, which compared screening outcomes using cervical cytology alone versus cytology plus HPV testing over two rounds, three years apart, as described in detail previously (Kitchener et al 2009 DOI: 10.1016/ S1470-2045(09)70156-1). The final analysis looked at 59 women in 3 time points (samples, n=177).
Data exclusions	Samples from six women (n=18) were excluded due to inadequate read coverage (< 10,000 reads per sample).
Replication	Not applicable
Randomization	Not applicable
Blinding	Authors were not blinded during analysis as the CIN levels were needed to separate the groups and perform statistical analyses between them.

Reporting for specific materials, systems and methods

We require information from authors about some types of materials, experimental systems and methods used in many studies. Here, indicate whether each material, system or method listed is relevant to your study. If you are not sure if a list item applies to your research, read the appropriate section before selecting a response.

Materials & experimental systems

n/a	Involved in the study
☒	☐ Antibodies
☒	☐ Eukaryotic cell lines
☒	☐ Palaeontology and archaeology
☒	☐ Animals and other organisms
☐	☒ Clinical data
☒	☐ Dual use research of concern
☒	☐ Plants

Methods

n/a	Involved in the study
☒	☐ ChIP-seq
☒	☐ Flow cytometry
☒	☐ MRI-based neuroimaging

Clinical data

Policy information about clinical studies

All manuscripts should comply with the ICMJE guidelines for publication of clinical research and a completed CONSORT checklist must be included with all submissions.

Clinical trial registration

The use of the ARTISTIC samples in this study have been approved by the NRES Committee Southeast Coast – Brighton and Sussex (Study title: Long-term follow-up of ARTISTIC cervical screening trial cohort) with the REC reference: 14/LO/0627 IRAS project ID: 153311.

Study protocol

IRAS project ID: 153311.

Data collection

Sixty-five women were selected from the ARTISTIC trial, which compared screening outcomes using cervical cytology alone versus cytology plus HPV testing over two rounds, three years apart, as described in detail previously (Kitchener et al 2009 DOI: 10.1016/S1470-2045(09)70156-1). The final analysis looked at 59 women in 3 time points (samples, n=177).

Outcomes

This longitudinal pilot study examines how CIN3 surgical excision impacts CVM composition, comparing differences between CIN3 progression, persistent hrHPV infection, and healthy controls. Its outcomes revealed that women scheduled for CIN3 surgical treatment had significantly higher levels of Lactobacillus spp-depleted CVMs compared to post-treatment. Lactobacillus gasseri emerged as a potential indicator of treatment recovery and post-treatment HPV clearance. Additionally, Sneathia amnii consistently appeared as a potential microbial biomarker for CIN3 development, exhibiting differential abundance in CIN3 pre-treatment specimens compared to post-treatment, persistently hrHPV+, and control groups. Conversely, higher proportions of Lactobacillus helveticus, Lactobacillus suntoryeus, and Lactobacillus vaginalis demonstrated a potential protective role against CIN3 development in women with persistent hrHPV infections.

Plants

Seed stocks

Not applicable

Novel plant genotypes

Not applicable

Authentication

Not applicable
